# Brain transcriptome atlases: a computational perspective

**DOI:** 10.1007/s00429-016-1338-2

**Published:** 2016-12-01

**Authors:** Ahmed Mahfouz, Sjoerd M. H. Huisman, Boudewijn P. F. Lelieveldt, Marcel J. T. Reinders

**Affiliations:** 1 Department of Radiology, Leiden University Medical Center, Leiden, The Netherlands; 2 Delft Bioinformatics Laboratory, Delft University of Technology, Delft, The Netherlands

**Keywords:** Brain atlases, Gene expression, Co-expression, Omics integration, Imaging genetics

## Abstract

The immense complexity of the mammalian brain is largely reflected in the underlying molecular signatures of its billions of cells. Brain transcriptome atlases provide valuable insights into gene expression patterns across different brain areas throughout the course of development. Such atlases allow researchers to probe the molecular mechanisms which define neuronal identities, neuroanatomy, and patterns of connectivity. Despite the immense effort put into generating such atlases, to answer fundamental questions in neuroscience, an even greater effort is needed to develop methods to probe the resulting high-dimensional multivariate data. We provide a comprehensive overview of the various computational methods used to analyze brain transcriptome atlases.

## Mapping gene expression in the brain

The mammalian brain is a complex system consisting of billions of neuronal and glia cells that can be categorized into hundreds of different subtypes. Understanding the organization of these cells, throughout development, into functional circuits carrying out sophisticated cognitive tasks can help us better characterize disease-associated changes. Advances in technology and automation of laboratory procedures have facilitated high-throughput characterization of functional neuronal circuits and connections at different scales (Pollock et al. 2014). For example, the Human Connectome Project maps the complete wiring of the brain using magnetic resonance imaging (Van Essen and Ugurbil 2012). Despite the importance of these imaging modalities in characterizing brain pathologies and development, it is imperative to analyze the molecular structure to gain a better mechanistic understanding of how the brain works. However, studying the molecular mechanisms of the brain has proved very challenging due to the unknown large number of cell types (Sunkin 2006).

The complexity of the brain is largely reflected in the underlying patterns of gene expression that defines neuronal identities, neuroanatomy, and patterns of connectivity. With 80% of the 20,000 genes in the mammalian genome expressed in the brain (Lein et al. 2007), characterizing spatial and temporal gene expression patterns can provide valuable insights into the relationship between genes and brain function and their role throughout neurodevelopment. Brain transcriptome atlases have proven to be extremely instrumental for this task.

Following earlier progress in other model organisms (Kim et al. 2001; Spencer et al. 2011; Milyaev et al. 2012), several projects have assessed gene expression in the mouse brain with various degrees of coverage for genes, anatomical regions, and developmental time-points (Sunkin 2006; Pollock et al. 2014). In rodents, the Gene Expression Nervous System Atlas (GENSAT) (Gong et al. 2003; Heintz 2004) and GenePaint (Visel et al. 2004) mapped gene expression in both the adult and developing mouse brain, while the EurExpress (Diez-Roux et al. 2011) and the e-Mouse Atlas of Gene Expression (EMAGE) (Richardson et al. 2014) focused on the developing mouse brain. Comparable atlases of gene expression in the human brain are far less abundant due to the challenges posed by difference in size between the human and mouse brain as well as the scarcity of post-mortem tissue. However, several studies have profiled the human brain transcriptome to analyze expression variation across the brain (Lonsdale 2013), expression developmental dynamics (Oldham et al. 2008; Colantuoni et al. 2011; Kang et al. 2011), and differential expression in the autistic brain (Voineagu et al. 2011), albeit in a limited number of coarse brain regions.

**Fig. 1:**
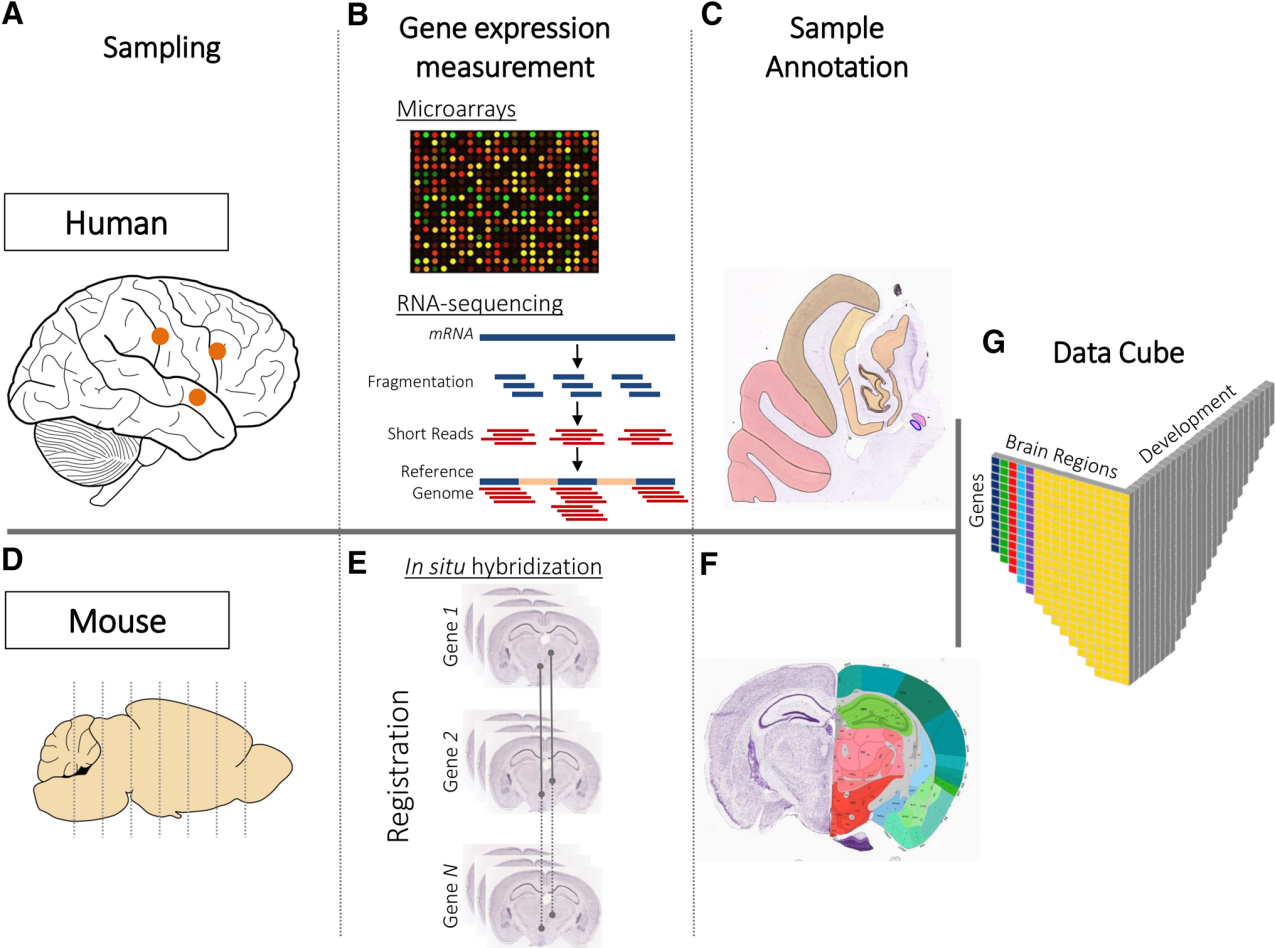
Spatially mapped gene expression in the mammalian brain. To map gene expression across the human and mouse brains, the Allen Institute for Brain Sciences followed two different strategies. In the human brain, samples covering all brain regions are extracted (**a**) and gene expression is measured using either microarray or RNA-sequencing (Hawrylycz et al. 2012; Miller et al. 2014b) (**b**). Accompanying histology sections and MRI scans are acquired to localize samples. Manual delineation of anatomical regions on the histology sections allowed for accurate sample annotation (**c**). In the mouse brain, gene expression is measured in coronal and sagittal sections using in situ hybridization (Lein et al. 2007) (**d**). Several slices covering the mouse brain are extracted per gene. Image registration methods are used to align the set of sections acquired for each gene to a common reference atlas (**e**). Anatomical regions are delineated on the reference atlas allowing for sample annotation (**f**). Data from the mouse and human atlases can be represented in a data matrix of three dimensions representing: genes, brain regions, and developmental stages (in case of the developmental atlases) (**g**)

The Allen Institute for Brain Science provides the most comprehensive maps of gene expression in the mouse and human brain in terms of the number of genes, the spatial-resolution, and the developmental stages covered (Pollock et al. 2014). Several atlases have been released which map gene expression in the adult and developing mouse brain (Lein et al. 2007; Thompson et al. 2014), the adult and developing human brain (Hawrylycz et al. 2012; Miller et al. 2014a), and the adult and developing non-human primate (NHP) brain (Bernard et al. 2012; Bakken et al. 2016); see Fig. 1. Sunkin et al. (2013) provides a complete review of the Allen Brain Atlas resources.

The availability of genome-wide spatially mapped gene expression data provides a great opportunity to understand the complexity of the mammalian brain. It provides the necessary data to decode the molecular functions of different cell populations and brain nuclei. However, the diversity of cell types and their molecular signatures and the effect of mutations on the brain remain poorly understood. For example, de novo loss-of-function mutations in autistic children have been shown to converge on three distinct pathways: synaptic function, Wnt signaling, and chromatin remodeling (Krumm et al. 2014; De Rubeis et al. 2014). Except for the synaptic role of autism-related genes, it is not clear how alternations in basic cell functions, such as Wnt signaling and chromatin remodeling, can result in the complex phenotype of autism spectrum disorders (ASD). A recent effort to map somatic mutations in cortical neurons using single-cell sequencing has shown that neurons have on average ~1500 transcription-associated mutations (Lodato et al. 2015). The significant association of these single-neuron mutations and genes with cortical expression indicates the vulnerability of genes active in human neurons to somatic mutations, even in normal individuals. The difference between these patterns in the normal and diseases brains remains unclear. Efforts to understand genotype-phenotype relationships in the brain face several challenges, including the complexity of the underlying molecular mechanisms and the poor definition of clinically based neurological disorders. In addition, the high-dimensionality of the data makes most studies underpowered to detect any associations. This is especially true in the case of testing genetic associations with phenotype markers, such as imaging measurements (Medland et al. 2014). A combination of efforts to map the genomic landscape of the brain and data-driven approaches can add to our understanding of the underlying genetic etiology of neurological processes and how they are altered in neurological disorders.

Several review articles provide extensive insights into the gene expression maps of the brain. French and Pavlidis (2007) provide a global overview of neuroinformatics, including ontology, semantics, databases, connectivity, electrophysiology, and computational neuroscience. Jones et al. (2009) give an overview on developing the mouse atlas, the challenges faced, the community reaction, limitations, and atlas usage examples, as well as the data mining tools provided by the Allen institute. Pollock et al. (2014) provide a detailed review of the technology and tools which are currently advancing the field of molecular neuroanatomy. Recently, Parikshak et al. (2015) illustrated the power of using network approaches to leverage our understanding of the genetic etiology of neurological disorders. Yet, a global overview of the computational methodologies applied to brain transcriptome atlases to increase our understanding of neurological processes and disorders remains missing.

In this review, we provide an overview of the computational approaches used to expand our understanding of the relationship between gene expression on one hand and the anatomical and functional organization of the mammalian brain on the other hand. We focus our discussion on spatial and temporal brain transcriptomes mapped by the Allen Institute for Brain Sciences. Nevertheless, we also discuss how the methods can be extended to epigenomes and proteomes of the brain and other human tissues. We describe the different computational approaches taken to analyze the high-dimensional data and how they have contributed to our understanding of the functional role of genes in the brain, molecular neuroanatomy, and genetic etiology of neurological disorders. Finally, we discuss how these methods can help solve some of the data-specific challenges, and how the integration of several data types can further our understanding of the brain at different scales, ranging from molecular to behavioral.

## Computational analysis of spatial and temporal gene expression data in the brain

Spatio-temporal transcriptomes of the brain pose several challenges due to their high-dimensionality. In this section, we identify the different types of approaches taken to analyze the spatially mapped gene expression data. We show the strengths of each approach and demonstrate how it has enriched neuroscience research. We divide the different methods into two categories. First, we describe a class of methods used to analyze the expression profile of gene(s) across different brain regions, cell types, and developmental stages. Second, we discuss methods focusing on the molecular organization and the genetic signature of the brain.

## Analyzing the expression patterns of genes in the brain

Mapping gene expression across the brain is very helpful in determining the neural function of a gene of interest by associating it with a specific brain region and/or developmental stage or in identifying genetic markers of those brain regions and developmental stages. Brain transcriptome atlases, such as the Allen Brain Atlases, provide useful information about the expression of a gene under “normal” conditions. Such information can be used to direct in-depth studies about a specific gene in biologically/clinically relevant cohorts. With the increasing number of genes implicated in neurological diseases as well as the realization that complex phenotypes of the brain likely result from the combined activity of several genes, a number of studies analyze gene sets rather than individual candidate genes. By studying the expression of a gene set rather than a single gene, neuroscientists are faced with a challenge on how to summarize this data to understand the relationship between genes and neuronal phenotypes.

### Gene expression visualization

**Fig. 2:**
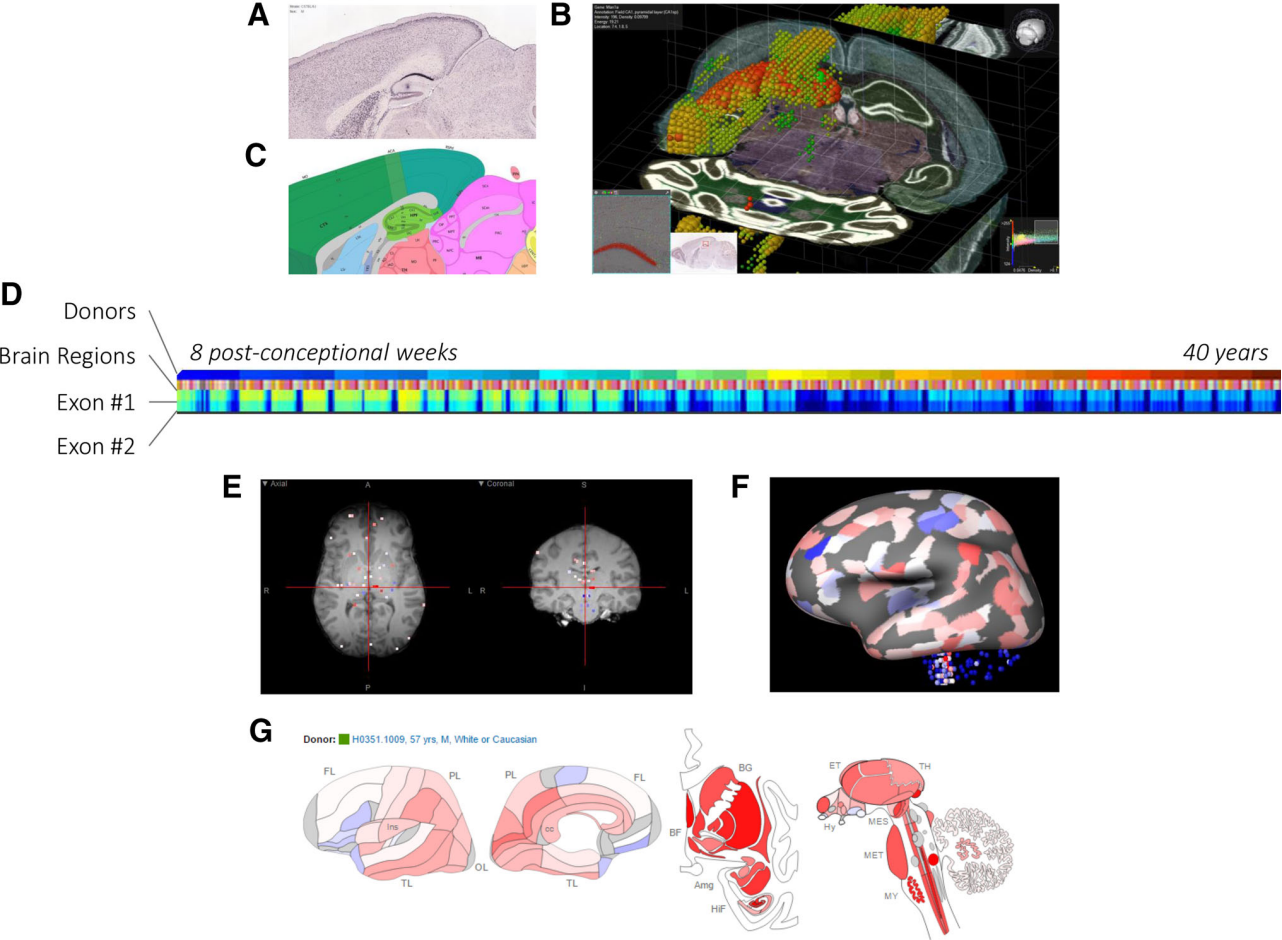
Gene expression visualization. Gene expression of spatially mapped samples can be visualized using several approaches. **a** Mouse gene expression data of the gene *Man1a* can be investigated using the original ISH sections. **b** BrainExplorer software allows visualization of the 3D expression volume with an overlay of the anatomical atlas and the ability to go back to the original high-resolution ISH section. **c** Simultaneously, viewing the ISH section and the corresponding atlas section helps in localizing gene expression to brain regions. **d** Heatmaps are commonly used to visualize gene expression. Expression of the two exons of the *NEUROD6* gene from the BrainSpan Atlas is visualized using a heatmap in which samples are ordered according to the age of the donor. **e** Samples from the Allen Human Brain Atlas are associated with coordinates of their location in the corresponding brain MRI. **f** Using the BrainExplorer, expression values of *Mecp2* can be mapped to an inflated white matter surface for better visualization of the cortex. **g** Alternatively, expression values can be mapped on an anatomical atlas of the human brain

High-throughput data visualization approaches can facilitate the exploration of complex patterns in multivariate high-dimensional gene expression data sets (Pavlopoulos et al. 2015). For example, heatmaps are commonly used to visualize gene expression levels across a set of samples using a two-dimensional false-color image (Fig. 2f). However, heatmaps are not ideal to represent brain transcriptomes, because they fail to capture the multivariate nature of the data (genes, samples, and time-points) and to represent the inherent spatial and temporal relationships between different brain regions and developmental stages, respectively. To acquire high-resolution gene expression maps, the Allen atlases of the developing and adult mouse brain rely of ISH images (Fig. 2a). The Brain Explorer 3D viewer (Lau et al. 2008) is an interactive desktop application that allows the visualization of the 3D expression of one or more genes with the possibility to link them back to the high-resolution ISH images (Sunkin et al. 2013) (Fig. 2b). ISH images can be synchronized between different genes and also with the anatomical atlas of the mouse brain (Fig. 2c), facilitating the analysis of a group of genes. For the adult and developing human atlases, the gene expression data (microarray or RNA-seq) are mainly visualized using heatmaps (Fig. 2d). In the adult human atlas, the expression data can also be visualized on top of the magnetic resonance images (Fig. 2e). The Brain Explorer 3D viewer is also used to visualize gene expression from cortical samples using an inflated cortical surface, a surface-based representation of the cortex that allows better representation of the relative locations of laminar, columnar, and areal features (Fig. 2f). In addition, gene expression can be mapped to an anatomical representation of the brain to facilitate interpretation (Fig. 2g). Ng et al. developed a method to construct surface-based flatmaps of the mouse cortex that enables mapping of gene expression data from the Allen Mouse Brain Atlas (Ng et al. 2010). Similarly, French (2015) developed a pipeline to map the expression of any gene from the Allen Human brain atlas to the cortical atlas built into the FreeSurfer software, which shall facilitate integration with medical imaging studies.

### Summary statistics and visualization-based methods

The early studies employing the Allen Brain Atlases used a variety of visualization and qualitative measurements to analyze the expression of gene sets associated with dopamine neurotransmission (Björklund and Dunnett 2007), consummatory behavior in the mouse brain (Olszewski et al. 2008), midbrain dopaminergic neurons (Alavian and Simon 2009), and changes in locomotor activity in the mouse brain (Mignogna and Viggiano 2010). Kondapalli et al. (2014) used a similar qualitative approach to analyze the expression of Na^+^/H^+^ exchangers (NHE6 and NHE9), which are linked to several neuropsychiatric disorders, in the adult and developing mouse brain atlases.

To provide better quantitative representations of the expression of gene sets, several studies relied on basic summary statistics, such as the mean and standard deviation. Zaldivar and Krichmar (2013) used summations to summarize the expression of cholinergic, dopaminergic, noradrenergic, and serotonergic receptors in the amygdala, and in neuromodulatory areas. By plotting the average expression of genes harboring de novo loss-of-function mutations identified by means of exome sequencing across human brain development, Ben-David and Shifman (2012a) identified two clusters with antagonistic expression patterns across development. In addition, spatio-temporal exonic expression in the BrainSpan atlas correlates inversely with the burden of deleterious de novo mutations identified by exome sequencing in autism, schizophrenia, or intellectual disability (Uddin et al. 2014). For genes mutated in autism, the inverse relationship was found to be strongest in prenatal orbital frontal cortex, highlighting the value of the BrainSpan atlas to associate genetic variation with specific brain regions and developmental stages. Dahlin et al. (2009) developed a custom score (expression factor) of gene expression in the mouse brain based on the ISH images of the Allen Mouse Brain Atlas. They computed the mean and the standard deviation of the expression factor to assess the global expression and heterogeneity of solute carrier genes, respectively. To deal with the qualitative ISH-based expression data from the Allen Mouse Brain Atlas, Roth et al. (2013) used a non-parametric representation of the data (using ranks instead of the raw expression values) to study the relationship between genes associated with grooming behavior in mice and 12 major brain structures.

Most of the studies analyzing gene expression in the brain focused on scores describing the expression of a gene or a gene set within each brain region of interest. Liu et al. (2014) proposed a characterization of the stratified expression pattern of sonic hedgehog (*Shh*), a classical signal molecule required for pattern formation along the dorsal–ventral axis, and its receptor *Ptch1*. Using a combination of differential expression, transcription factor motif analysis, and CHIP-seq, they identified the role of *Gata3*, *Fox2*, and their downstream targets in pattern formation in the early mouse brain. These results illustrate the power of characterizing complex expression patterns across the brain rather than solely summarizing the expression of each gene within individual brain regions.

**Table:** 

Box1 | **Gene Sets** Complex biological functions and disorders usually involve several rather than a single gene. Gene sets are groups of genes that share common biological functions and that can be defined either based on prior knowledge (e.g. about biochemical pathways or diseases) or experimental data (e.g. transcription factor targets identified using CHIP-seq). Gene set databases organize existing knowledge about these groups of genes by arranging them in sets that are associated with a functional term, such as a pathway name or a transcription factor that regulates the genes. Gene sets can be classified into 5 types:
**Gene Ontology (GO)** The Gene Ontology project (Ashburner et al. 2000) developed three hierarchically structured vocabularies (ontologies) that describe gene products in terms of their associated *biological processes*, *cellular components* and *molecular functions*. Genes annotated with the same GO term(s) constitute a gene set.
**Biological Pathways** Biological pathways are networks of molecular interactions underlying biological processes. Pathway databases, such as Kyoto Encyclopedia of Genes and Genomes (KEGG) (Ogata et al. 1999) and REACTOME (Croft et al. 2014), catalog physical entities (proteins and other macromolecules, small molecules, complexes of these entities and post-translationally modified forms of them), their subcellular locations and the transformations they can undergo (biochemical reaction, association to form a complex and translocation from one cellular compartment to another).
**Transcription** Transcription databases include information on regulation of genes by transcription factors (TFs) binding to the DNA, or post-transcriptional regulation by microRNA binding to the mRNA. Determining these physical interactions can be done either *in silico* using computational inference (motif enrichment analysis) or using experimental data (such as CHIP-seq and microRNA binding data). For the motif enrichment analysis, position weight matrices (PWMs) from databases TRANSFAC (Matys et al. 2006) and JASPER (Portales-Casamar et al. 2010) can be used to scan the promoters of genes in the region around the transcription factor start site (TSS). CHIP-seq data, such as the large collection of experiments from the Encyclopedia of DNA Elements (ENCODE) project (Bernstein et al. 2012b) and the Roadmap Epigenomics consortium (Consortium 2015a), is used to identify genes targeted by the TFs. Similarly, microRNA targets can be extracted from databases such as TargetScan (Lewis et al. 2003).
**Cell-type markers** Cell type-specific transcriptional data provide a very rich source of cell type marker genes. Genes are identified as a cell type marker if they are up-regulated in one cell population compared to other cell populations. Several studies have used microarrays and RNA-seq to profile the transcriptome of a number of neuronal cell types (Cahoy et al. 2008; Zhang et al. 2014). Recently, studies are using single-cell sequencing to precisely capture the transcriptome of individual neuronal cells (Darmanis et al. 2015; Zeisel et al. 2015).
**Disease** Genes can be grouped into sets based on their association to the same diseases. Public databases, such as OMIM (2015a) and DisGeNet (Pinero et al. 2015), contains curated information from literature and public sources on gene-disease association. Another source to obtain disease-related gene sets is by identifying genes harboring variants identified using GWAS (Simón-Sánchez and Singleton 2008; Welter et al. 2014), exome-sequencing (2015b), or whole-genome sequencing.

### Identifying genes with localized expression patterns

The complexity of the brain implies that genes are involved in more than one function and that their function is region- or cell-type-specific. Neuronal cell types have been classically defined using cell morphology, electrophysiological and connectivity properties. Similarly, classical neuroanatomy identifies regions based on their cyto-, myelo-, or chemo-architecture. Genomic transcriptome measurements provide an alternative route to define functional cell types and brain regions based on their genetic makeup.

Several studies have analyzed the ISH-based gene expression images of the Allen Mouse Brain Atlas to identify cell-type-specific genes and genes with localized gene expression. Loerch et al. (2008) studied the localization of age-related gene expression changes in different neuronal cell types in the mouse and human brains. At the brain region level, David and Eddy (2009) developed ALLENMINER, a tool that searches the Allen Mouse Brain Atlas for genes with a specific expression pattern in a user-defined brain region. At a finer scale, Kirsch et al. (2012) described an approach to identify genes with a localized expression pattern in a specific layer of the mouse cerebellum. They represented each ISH image (gene) using a histogram of local binary patterns (LBP) at multiple-scales. Predicting the localization of gene activity to each of the four cerebellar layers is done using two-level classification. First, they used a support vector machine (SVM) classifier to assign a cerebellar layer to each image and then used multiple-instance learning (MIL) to combine the resulting image classification into gene classification. Similarly, to identify cell-type specific genes, Li et al. (2014) used scale-invariant feature transform (SIFT) features of the ISH images. They further classified genes, using a supervised learning approach (regularized learning), based on their expression in different brain cell types. Zeng et al. (2015) compared two models to extract features from the ISH images of the developing mouse brain atlas to train a classification model to annotate gene expression patterns in brain structures. In one approach, they used SIFT features and the bag-of-words approach to represent the expression of each gene across the entire brain. In addition, they used a transfer learning approach by training a deep convolutional neural network on natural images to extract useful features from the ISH images. Their results show a superior performance for the deep convolutional neural network, indicating the applicability of transfer learning from natural to biological images (Zeng et al. 2015).

Ramsden et al. (2015) studied the molecular components underlying the neural circuits encoding spatial positioning and orientation in the medial entorhinal cortex (MEC). They developed a computational pipeline for automated registration and analysis of ISH images of the Allen Mouse Brain Atlas at laminar resolution. They showed that while very few genes are uniquely expressed in the MEC, differential gene expression defines its borders with neighboring brain structures, and its laminar and dorso-ventral organization. Their analysis identifies ion channel-, cell adhesion- and synapse- related genes as candidates for functional differentiation of MEC layers and for encoding of spatial information at different scales along the dorso-ventral axis of the MEC. Finally, they reveal laminar organization of genes related to disease pathology and suggest that a high metabolic demand predisposes layer II to neurodegenerative pathology.

### Spatial and temporal gene co-expression

Genes with similar expression patterns over a set of samples are said to be co-expressed and are more likely to be involved in the same biological processes (guilt by association) (Stuart et al. 2003). Applying the same approach to brain transcriptomes can identify co-expressed genes based on their spatial and/or temporal expression across the brain. This can serve as a powerful tool to characterize genes with respect to their context-specific functions. In addition, co-expression has been used to assess the quality of RNA-seq data, such as the BrainSpan atlas, by modeling the effects of noise within observed co-expression (Ballouz and Gillis 2016a).

**Table:** 

Box 2 | **Dimensionality reduction**
The high dimensionality of transcriptomes, and other biological data (e.g. proteomes, epigenomes, etc.), provides a challenge for visualization as well as for selecting informative features for clustering and classification. Dimensionality-reduction approaches aim at finding a smaller number of features that can adequately represent the original high dimensional data in a lower dimensional space. The conventional principal component analysis (PCA) is the most commonly used dimensionality reduction method. Despite its utility, PCA can only capture linear rather than non-linear relationships, which are inherent in many biological applications. Several non-linear dimensionality reduction techniques have been proposed (e.g. Isomap (Tenenbaum et al. 2000)), see (Lee and Verleysen 2005) for an extensive review. The t-distributed stochastic neighbor embedding (t- SNE) method (Maaten and Hinton 2008) has been widely used to visualize biological data in two dimensions by preserving both the global and local relationships between the data points in the high-dimensional space (Saadatpour et al. 2015).

**Fig. 3:**
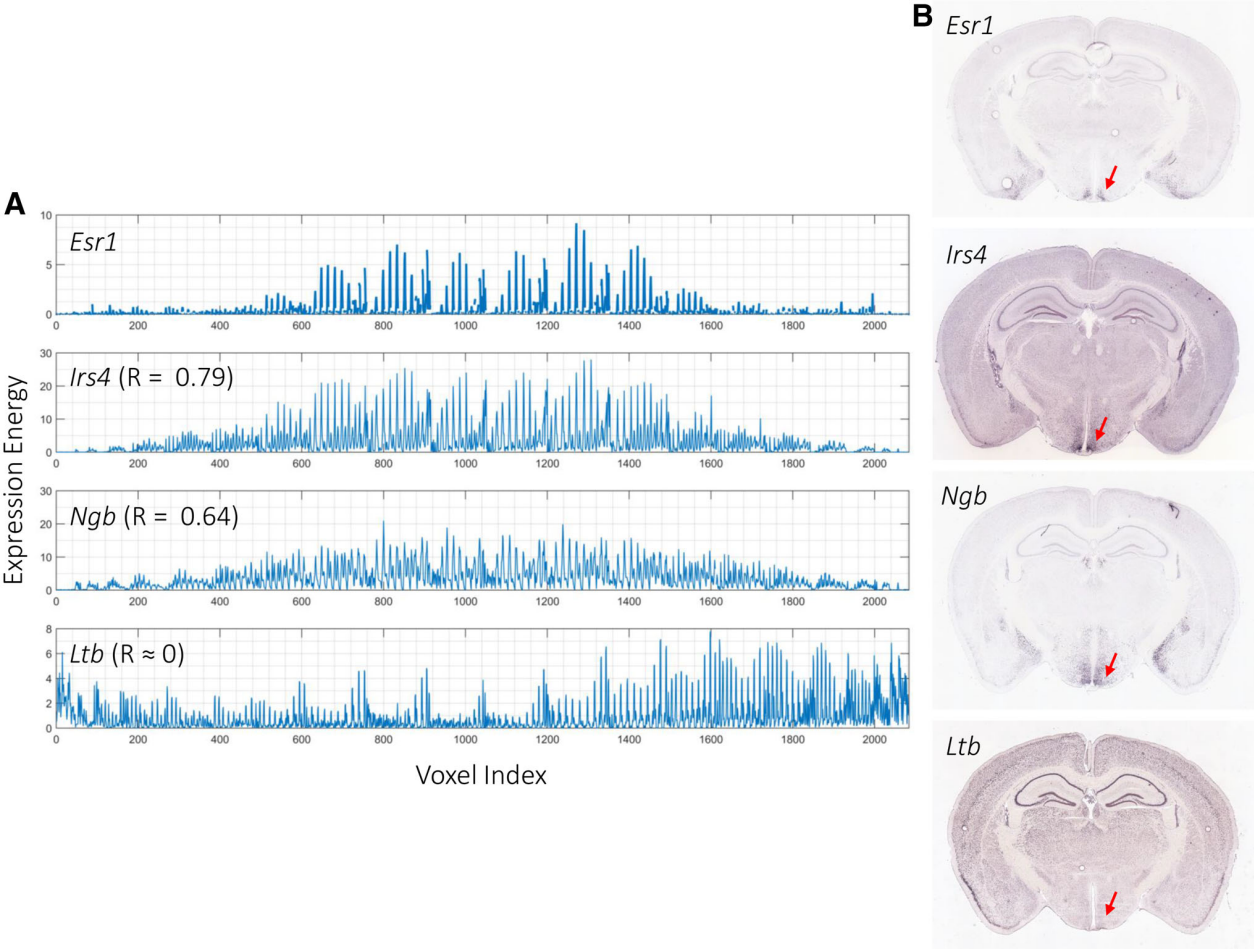
Spatial gene co-expression in the mouse brain. **a** Expression energy profiles of voxels in the hypothalamus region of the mouse brain using the same linear ordering. The estrogen receptor alpha (*Esr1*) gene shows high expression in the hypothalamus. The expression patterns of *Irs4* and *Ngb* are highly correlated with that of *Esr1* (*R* = 0.79 and *R* = 0.64, respectively). On the other hand, the expression pattern of *Ltb* is not correlated with that of *Esr1* (*R* = 8.01 × 10^−4^). Correlation is calculated using Pearson correlation. **b** *Esr1* and its highly correlated genes (*Irs4* and *Ngb*) are highly expressed in the hypothalamus (*red arrow*), while *Ltb* is not

Several similarity/distance measurements have been used to characterize the similarity in spatial/temporal expression patterns between a pair of genes. Of these, correlation-based measures are mostly used to assess gene co-expression patterns across the brain. NeuroBlast is a search tool developed by the Allen Institute for Brain Sciences to identify genes with a similar 3D spatial expression to that of a gene of interest in a given anatomical region, based on Pearson correlation (Hawrylycz et al. 2011). Figure 3a shows an example of the obtained correlations of estrogen receptor alpha (*Esr1*) in the mouse hypothalamus. The ISH sections in Fig. 3b show that correlation can effectively be used to identify genes’ functional association with *Esr1*. For example, the top correlated gene to *Esr1* in the hypothalamus is insulin receptor substrate 4 (*Irs4*), a target gene of *Esr1* associated with sex-specific behavior (Xu et al. 2012). NeuroBlast was used to identify genes with a similar expression profile to *Wnt3a*, a ligand in the Wnt signaling pathway, in the developing mouse brain and identified eight Wnt signaling genes among the top correlated genes (Thompson et al. 2014). Using Spearman correlation coefficient, French et al. analyzed gene-pairs with positive and negative co-expression in the mouse brain. By focusing on genes with a strong negative correlation, they showed that variation in gene expression in the adult normal mouse brain can be explained as reflecting regional variation in glia to neuron ratios, and is correlated with degree of connectivity and location in the brain along the anterior–posterior axis (French et al. 2011). Tan et al. (2013) extended the analysis to the adult human brain and identified conserved co-expression patterns between the mouse and the human brain. To characterize the role of *SNCA*, a gene harboring a causative mutation for Parkinson’s disease, Liscovitch and French (2014) analyzed the co-expression relationships of *SNCA* in the adult and developing human brain. They identified a negative spatial co-expression between *SNCA* and interferon-gamma signaling genes in the normal brain and a positive co-expression in post-mortem samples from Parkinson’s patients, suggesting an immune-modulatory role of *SNCA* that may provide insight into neurodegeneration. Another example is given by Bernier et al. (2014), in which the developing human, macaque, and mouse brain atlases were used to analyze the expression and co-expression patterns of *CHD8*, one of the key autism-associated genes. Their analysis showed that *CHD8* was expressed throughout cortical and sub-cortical structures at the early prenatal ages and that expression decreased through development. In addition, they showed a significant enrichment of autism-candidate genes among genes with correlated temporal patterns to *CHD8* in the BrainSpan atlas.

**Table:** 

Box 3 | **Clustering**
Clustering is the unsupervised learning process of identifying distinct groups of objects (clusters) in a dataset (Duda et al. 2000). There are two main types of clustering: hierarchical and partitional. Hierarchical clustering algorithms start by calculating all the pair-wise similarities between samples and then building a dendrogram by iteratively grouping the most similar sample pairs. By cutting the tree at an appropriate height, the samples are grouped into clusters. On the other hand, partitional clustering optimizes the number of simple models to fit the data. Examples of partitional clustering include k-means, Gaussian mixture models (GMMs), density-based clustering, and graph-based methods.
In order to cluster the samples hierarchically, all the pair-wise similarities between sample S_i_ and S_j_ are calculated. Samples are then grouped iteratively based on the calculated similarities (grouping the most similar first). Once the full dendrogram is built, a cut-off (dashed line) is used to group samples into groups. For k-means we set the number of clusters based on the data heatmap. K-means groups samples by minimizing the within-cluster sum of square distances between each point in the cluster and the cluster center.

Gene co-expression can serve as a very powerful tool for in silico prediction and prioritization of disease genes, by identifying genes with similar expression pattern to known disease genes. Piro et al. (2010) described a candidate gene prioritization method using the Allen Mouse Brain Atlas. They showed that the spatial gene-expression patterns can be successfully exploited for the prediction of gene–phenotype associations by applying their method to the case of X-linked mental retardation. By extending their methods to the human brain atlas, they showed that spatially mapped gene expression data from the human brain can be employed to predict candidate genes for Febrile seizures (FEB) and genetic epilepsy with febrile seizures plus (GEFS^+^) (Piro et al. 2011). Both examples illustrate the power of using computational approaches to prioritize disease genes before carrying out empirical analysis in the lab.

In measuring gene co-expression, correlation-based methods are not specific to spatially mapped expression data and do not fully model the complexity of the brain transcriptomes. To identify gene-pairs with similar expression patterns in the adult mouse brain based on the ISH images, Liu et al. (2007) compared three image similarity metrics: a naïve pixel-wise metric, an adjusted pixel-wise metric, and a histogram- row-column (HRC) metric. They showed that HRC performs better than voxel-based methods, indicating the superiority of methods that capture the local structure in spatially mapped data. Miazaki and Costa (2012) used Voronoi diagrams to measure the similarity of the density distribution between gene expressions in the adult mouse brain. Inspired by computer vision algorithms, Liscovitch et al. (2013) used the similarity of scale-invariant feature transform (SIFT) descriptors of the ISH images of the mouse brain to predict the gene ontology (GO) labels of genes.

**Table:** 

Box 4 | **Classification**
Classification is a supervised learning process of labeling unseen objects (test set) given a set of labeled objects (training set) (Duda et al. 2000). Classification approaches can be divided into Bayesian methods and prediction error minimization methods. The former group is based on Bayesian decision theory and uses statistical inference to find the best class for a given object. Bayesian methods can be further divided into parametric classifiers (e.g nearest-mean classifier and Hidden Markov Model) and non-parametric classifiers (e.g. Parzen window or k-nearest neighbor classifier). Alternatively, classifiers can be designed to minimize a measure of the prediction error. Well-known classifiers in this category include regression classifiers (e.g. Lasso regression), support vector machines, decision trees and artificial neural networks. Neural networks (in particular Deep Learning), have become very successful in solving problems in a wide range of applications, including bioinformatics (Xiong et al. 2014; Alipanahi et al. 2015; Engelhardt and Brown 2015).
A low dimensional embedding of the samples is generated using two features (genes). A Baysian Classifier assigns each sample to one of the two classes (Diseases or Healthy) based on statistical inference. A prediction error-minimization classifier updates the classification boundary (dashed line) based on the prediction error and terminates when a certain criterion is met.

### Gene co-expression networks

As we have shown, the guilt by association paradigm has been successfully employed to identify pairs of spatially co-expressed genes sharing a neuronal function, based on various similarity measures. To extend the co-expression analysis of gene-pairs, clustering and network-based approaches can be used to identify molecular interaction networks of a group of genes that signal through similar pathways, share common regulatory elements, or are involved in the same biological process. Co-expression networks avoid the problem of relying on prior knowledge, such as protein–protein interactions and pathway information, which are valuable but incomplete. Gene co-expression networks have heavily been used to identify disrupted molecular mechanisms in cancer (Chuang et al. 2007; Yang et al. 2014) and aging (van den Akker et al. 2014).

Hierarchical clustering is a widely used unsupervised approach to identify groups of co-expressed genes across a set of samples. Using hierarchical clustering, Gofflot et al. (2007) identified the functional networks of nuclear receptors based on their global expression across different regions of the mouse brain. By focusing on subsets of brain structures involved in specialized behavioral functions, such as feeding and memory, they elucidated links between nuclear receptors and these specialized brain functions that were initially undetected in a global analysis. Dahlin et al. (2009) used hierarchical clustering to explore potential functional relatedness of the solute carrier genes and anatomic association with brain microstructures.

Another approach to unsupervised clustering is to use gene co-expression relationships to construct a co-expression network where nodes are genes and edges represent the similarity of the expression profile of those genes. Weighted gene co-expression network analysis (WGCNA) (Zhang and Horvath 2005) is a commonly used method to construct modules of co-regulated genes based on the topological overlap between genes in a weighted co-expression network. WGCNA has widely been used to identify transcription networks in the mammalian brain. Oldham et al. (2006) demonstrated the first utility of WGCNA to examine the conservation of co-expression networks between the human and chimpanzee brains. They found that module conservation in cerebral cortex is significantly weaker than module conservation in sub-cortical brain regions, which is in line with evolutionary hierarchies. WGCNA has been applied to identify modules of co-regulated genes in the developing and adult human brain transcriptomes (Kang et al. 2011; Hawrylycz et al. 2012), the developing rhesus monkey brain (Miller et al. 2013), the developing mouse brain (Thompson et al. 2014), and the prenatal human cortex (Miller et al. 2014a), see Fig. 3b. The methods provide a valuable insight into the molecular organization of the brain by identifying modules reflecting primary neural cell types and molecular functions. For example, modules constructed based on the prenatal human cortex correspond to cortical layers and age, while no areal patterning was observed (Miller et al. 2014a). In addition, WGCNA was used to identify a set of 32 functionally and anatomically distinct modules of genes with highly reproducible gene expression patterns across six human brains (Hawrylycz et al. 2015). There are numerous technical considerations to considere while constructing co-expression networks that go beyond the scope of this review (Allen et al. 2012; Ballouz et al. 2015). To analyze regional specificity of co-expression networks in the adult human brain, Myers et al. (2015) analyzed the modularity of a given gene set in region-specific co-expression networks. The developed method was used to compare networks that are constructed using expression data from a large sample size, but coarse neuroanatomical data set (Gibbs et al. 2010) to region-specific networks derived from the Allen Human Brain Atlas.

**Table:** 

Box 5 | **Co-expression Measurements** Gene co-expression is widely used for functional annotation, pathway analysis, and the reconstruction of gene regulatory networks. Co-expression measurements assess the similarity between a pair of gene expression profiles by detecting bivariate associations between them. These co-expression measurements can be summarized in five categories (Kumari et al. 2012; Allen et al. 2012; Song et al. 2012; Wang et al. 2014):
**Correlation** The most widely used co-expression measure is *Pearson correlation*, due to its straightforward conceptual interpretation and computational efficiency. However, Pearson correlation can only capture linear relationships between variables. Alternatively, Spearman correlation is a nonparametric measure of non-linear associations. Other correlation-based methods include Renyi correlation, Kendall rank correlation, and bi-weight mid-correlation.
**Partial correlation** Partial correlation is used to measure direct relationships between a pair of variables, excluding indirect relationships. Based on Gaussian graphical models, partial correlations infer conditional dependency as the non-zero entries in the precision matrix (the inverse of the covariance matrix).
**Mutual-Information** Mutual information-based methods measure general statistical dependence between two variables. Based on information theory, mutual information does not assume monotonic relationships and hence can capture non-linear dependencies.
**Other measures** Euclidian distance; Cosine similarity; Kullback-Leibler divergence; Hoeffding’s D, distance covariance, and probabilistic measures (as used in Baysian networks).

### Co-expression of disease-related genes

**Fig. 4:**
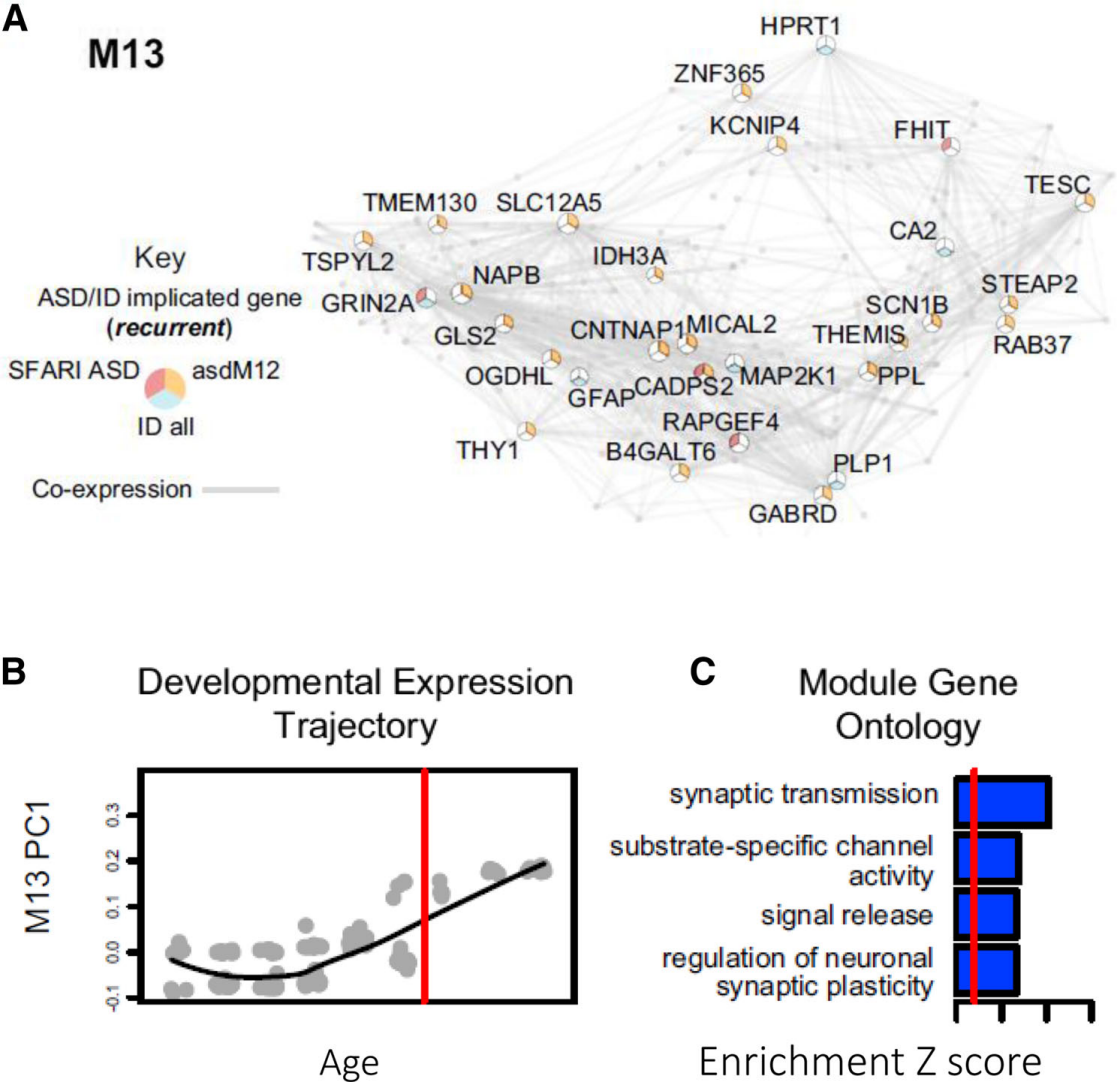
Gene co-expression networks. **a** Module M13 of co-expressed genes from Parikshak et al. (2013) (reprinted from Parikshak et al. Parikshak et al. 2013, Copyright (2016), with permission from Elsevier.). The shown module is significantly enriched in autism-related genes. The shown network comprises the top 200 connected genes (highest correlation) and their top 1000 connections in the subnetwork (also ordered on correlation). Genes are labeled if they are members of relevant gene sets. **b** Pattern of gene expression of genes in the shown module is summarized using the first principal component (eigengene). The *red line* indicates birth. **c** Gene Ontology terms enriched in the shown module. The *blue bars* indicate relative enrichment compared to all cortex-expressed genes in terms of *Z* score. The *red line* indicates *Z* = 2

Complex neuropsychiatric and neurological disorders involve dysregulation of multiple genes, each conferring a small but incremental risk, which potentially converge in deregulated biological pathways or cellular functions. Using genome-wide association studies (GWAS), exome sequencing, and whole-genome sequencing (WGS), hundreds of variants have been linked to complex neurological disorders, such as autism (Iossifov et al. 2012; Neale et al. 2012; O’Roak et al. 2012; Sanders et al. 2012; Dong et al. 2014; De Rubeis et al. 2014), schizophrenia (Fromer et al. 2014; Ripke et al. 2014), Migraine (Freilinger et al. 2012), and Alzheimer’s (Bettens et al. 2013; Zhang et al. 2013). With the increasing numbers of samples included in these studies, the number of variants associated to each disease is set to increase (Krumm et al. 2014). Gene co-expression networks provide a framework to identify the underlying molecular mechanisms on which these variants converge. Ben-David and Shifman (2012b) analyzed co-expression networks of genes affected by common and rare variants in autism using WGCNA. Menashe et al. (2013) used the cosine similarity of expression profiles to build a co-expression network of autism-related genes in the mouse brain. Both studies provide an important link between gene networks associated with autism and specific brain regions. However, for neurodevelopmental disorders, such as autism and schizophrenia, it is more beneficial to study when and where implicated genes are expressed during brain development. Gulsuner et al. (2013) studied the transcriptional co-expression of genes harboring de novo mutations in schizophrenia patients using the BrainSpan atlas of the Developing Human Brain. Parikshak et al. (2013) used WGCNA to identify modules of co-expressed genes during human brain development using the BrainSpan atlas. They identified modules with significant enrichment in autism-related genes (Fig. 4). Willsey et al. (2013) used the BrainSpan atlas to generate co-expression networks around nine genes harboring recurrent de novo loss-of-function mutations in autism probands. Mahfouz et al. (2015b) used a combination of differential expression and genome-wide co-expression analysis to identify shared pathways among autism-related genes. To assess the functional convergence of distinct sets of genetic variants, Ballouz and Gillis (2016b) analyzed the connectivity of autism-candidate genes within a co-expression network constructed from the BrainSpan atlas. Their results show that gene sets with a higher proportion of burden genes exhibit higher interconnectivity, indicating stronger functional associations.

Using gene co-expression networks to study relationships between disease-related genes is a valuable approach to understand disease mechanisms. In addition, using networks facilitates the integration of different types of interactions between genes, including but not limited to: co-expression, protein–protein interactions, and literature-based interactions. This can be very useful to our understanding of the etiologies of complex neurological diseases at different levels. In a recent study, Hormozdiari et al. (2015) integrated gene co-expression based on the BrainSpan atlas and PPI networks to identify networks of genes related to autism and intellectual disability. For a review on using gene networks to investigate the molecular mechanisms underlying neurological disorders, we refer to Gaiteri et al. (2014) and Parikshak et al. (2015).

**Table:** 

Box 6 | **Co-expression Networks** Gene co-expression networks provide a framework to uncover the molecular mechanisms underlying biological processes based on gene expression data. A co-expression network consists of nodes to represent genes and edges to encode the co-expression between two genes. A weighted network is a network in which the edges have continuous values to indicate the strength of co-expression. Networks with binary edges (an edge either exists or not) are termed binary networks. Analysis of co-expression networks can be summarized in four main steps:
**Network Construction** The first step in building a co-expression network is to construct a similarity matrix, by quantifying the similarity between the expression profiles of each pair of genes (i.e. co-expression). Several methods to measure gene co-expression are discussed in Box 5. For non-regularized estimations of co-expression, all off-diagonal elements of this similarity matrix will be nonzero. We can take these similarities as edge weights in the network, but that will give a fully connected network (each gene is connected to each gene). An additional step can be to threshold the similarity matrix, either to prune edges, or to binarize (absent/present) the similarities to obtain an adjacency matrix. In the latter case, pairs of genes with co-expression values above a threshold will be connected in a binary network. In the weighted gene co-expression network analysis (WGCNA) framework the similarity matrix undergoes a power transformation and a weight diffusion step, to optimize the topological properties and stability of the network (Zhang and Horvath 2005).
**Network Characterization** The obtained networks can be analyzed in a number of ways. Topological measures characterize the structure of the network, and quantify the importance of genes in their network context. These measures have been extended to weighted networks (Zhang and Horvath 2005), and can capture topology on different levels of scale (Hulsman et al. 2014). Sets of networks can also be aligned and compared (Przulj 2007; Hayashida and Akutsu 2010; Fionda 2011). Network comparison can be used either to assess changes between different conditions, or to replicate a network in an independent dataset for validity assessment.
**Module Identification** To interpret a network, it can be divided into sub-networks, or gene modules. To do this, the network edges are often treated as similarities in a clustering approach (see Box 3). Alternatively, graph properties, such as topological overlap or modularity, can be used to divide a network into modules (Blondel et al. 2008).
**Module Characterization** Finally, modules can be characterized using a wide range of approaches. The expression profile of genes within the same module can be summarized using the average or the first principle component (also called eigengene (Oldham et al. 2006)). Alternatively, one can characterize a module according to its hub genes: the genes with the largest number of connections within the module. Another option is to assess the association of a module to external data by testing statistical enrichment in various gene sets (see Box 1 for different types of gene sets). In addition, modules can be characterized based on changes between conditions (e.g. health and disease) in their summary statistics (average expression profile), their topological measures (inter-connectivity), or the number of differentially-expressed genes they include.

## Analyzing genetic signature of brain regions

Spatially mapped gene expression data allow for the exploration of neuroanatomy from a molecular point of view. Individual genes with spatially differential expression have long been used to define the structural organization of the brain and to break it down into regions and sub-regions. Genes have also been used to identify different classes of neuronal cell types. Studying the “genetic signature” of different brain regions can be useful for a multitude of applications. Spatially mapped gene expression data allow for the analysis of the similarity between brain regions in terms of their expression profiles. Regions sharing an expression profile are likely to be involved in the same neuronal functions or be part of the same neuronal circuit. Moreover, studying the expression profiles of functionally and anatomically connected structures provides valuable insights into the molecular basis of brain connectivity.

### Spatial and temporal similarity of regional gene expression patterns

Each of the Allen Brain Atlases assigns a spatial location and a time point to each sample, allowing the exploration of the structural organization of the brain based on spatial and temporal similarities between different brain regions across the expression of thousands of genes. The Anatomic Gene Expression Atlas (AGEA) is a Web-based tool to calculate voxel-wise correlations based on gene expression in the adult and developing mouse brain atlases (Ng et al. 2009). To show the value of using the similarity of gene expression patterns to study anatomical organization, Dong et al. (2009) used AGEA to identify three distinct functional domains in the CA1 region of the mouse hippocampus. Hawrylycz et al. (2010) used AGEA to show that a consistent expression-based organization of areal patterning in the mouse cortex exists when clustered on a laminar basis. Using a combination of voxel–voxel similarities in gene expression (AGEA) and gene–gene similarities in expression patterns (NeuroBlast), Wagner et al. (2016) identified transcriptional markers of the mouse habenula as well as its subnuclear organization. In contrast to methods identifying regional markers by analyzing one gene at a time (Ramsden et al. 2015), using correlations between voxels (AGEA) and genes (NeuroBlast) simultaneously, such as (Dong et al. 2009; Wagner et al. 2016), reveals the transcriptomic–anatomic organization of brain areas.

Voxel correlation maps, such as those obtained by AGEA, can be used to cluster the mouse brain voxels into regions with similar gene expression profile. To analyze whether anatomically delineated regions, as defined classically, can also be distinguished based on their expression profile, Bohland et al. (2010) clustered the adult mouse brain voxels based on the similarity of their expression profiles. Using k-means clustering, they showed that their parcellations are quantitatively similar to the classically defined neuroanatomical atlas. These results show that the spatially mapped gene expression data can be very valuable in identifying the molecular basis of brain organization. Similarly, Goel et al. (2014) used a combination of dimensionality reduction and spectral clustering to investigate the correspondence between spatial clusters of gene expression and human brain anatomy.

To identify which genes are responsible for brain organization, Ko et al. (2013) used a similar approach to cluster brain voxels based on their expression of gene markers of different cell types. Their results show that the neuroanatomical boundaries within a mouse brain can be defined by the clustering of only 170 neuron-specific genes. To identify the driving mechanism of spatial co-expression of genes in the brain, Grange et al. (2014) modeled co-expression patterns based on the spatial distribution of underlying cell types. Their model can be used to estimate cell-type specific maps of the mouse brain and to identify brain regions based on their genetic signatures. The model proposed in (Grange et al. 2014) was used to estimate the similarity between the expression profiles of two cliques of two cliques of co-expressed autism genes (Menashe et al. 2013) and the spatial distribution of cell types (Grange et al. 2015).

The temporal dynamics of gene expression patterns of brain regions, throughout brain development, have been considered in several studies. To understand gene expression specialization of mouse brain regions during development, Liscovitch and Chechik (2013) assessed the dissimilarities between brain regions based on gene expression and how these changeover time. Their results suggest an hourglass pattern, with high dissimilarity early in development that decreases to reach a minimum at birth after which it increases again. Using differential expression among regions of the human cortex at each development stage, Pletikos et al. (2014) also reported a highly similar temporal hourglass pattern of dissimilarity between brain regions. Another study by Mahfouz et al. (2014) analyzed the similarity between gene expression patterns of brain regions during human development. Using a network-based approach, they characterized the topology of the connectivity network of autism-related genes across development.

### Gene expression and brain connectivity

Another way to study brain organization and function is to consider brain connectivity. Brain connectivity has been linked to many neurological disorders, such as ischemic stroke, autism, and schizophrenia. The relationship between gene expression and neuronal connectivity has long been studied in model organisms, such as *Caenorhabditis elegans*, to identify genes involved in synaptogenesis and axon guidance (Varadan et al. 2006; Kaufman et al. 2006; Baruch et al. 2008).

Zaldivar and Krichmar (2013) used the Allen mouse brain atlas to study the expression patterns of neurotransmitters in the brain. Since the expression of a transmitter must be coupled with the expression of appropriate receptors in the postsynaptic target, they have also analyzed the expression of receptors in target regions. This study shows that known neurobiological concepts can be seen back in the Allen brain atlas. To take it one step further, French and Pavlidis (2011) and Wolf et al. (2011) analyzed the relationship between gene expression similarity of brain regions and their connectivity. Both studies used the Allen mouse brain atlas to calculate the similarity in gene expression between different regions and the neural connectivity data of the rat brain from the Brain Architecture Management System (BAMS) (Bota and Swanson 2010). Genes involved in brain development and neurodevelopmental disorders, such as autism, showed strong correlations with anatomical connectivity patterns.

With the recent availability of the Allen mouse connectivity atlas, it has become possible to study the relationship between gene expression and brain connectivity within the same species. Rubinov et al. (2015) used a multivariate dimensionality reduction approach, partial least squares, to explore the association between gene expression and connectivity in the mouse brain. Rather than assessing the correlation between the gene expression similarity and connectivity, Ji et al. (2014) and Fakhry and Ji (2014) set out to predict connectivity based on gene expression patterns. By analyzing highly connected regions (i.e., hubs) in the mouse brain, Fulcher and Fornito (2016) showed that these hubs are more likely to interconnect with each other and are more likely to be transcriptionally similar. More interestingly, the genes with the highest contribution to the transcriptional similarity between hubs are involved in regulating the synthesis and metabolism of ATP, which is the primary energy source for neural activity.

### Integrating gene expression and brain imaging data

The anatomical locations of samples in the Allen Human Brain Atlas have been indicated in the MRI scans of each of the six donor brains. These scans have been mapped to the Montreal Neurological Institute (MNI) standardized coordinate space, allowing for easy integration with other imaging studies. Rizzo et al. (2014) tested the predictive power of mRNA transcription maps extracted from the Allen Human Brain Atlas to predict in vivo protein distributions acquired using positron emission tomography (PET) imaging. By analyzing genes involved in two neurotransmission systems with different regulatory mechanisms, they showed that in vivo protein distributions can be predicted from mRNA transcription maps when expression is being regulated translationally instead of posttranscriptionally. In another study, mRNA data from the Allen Human Brain Atlas were used to estimate the specific and non-displaceable components of PET radioligands for brain receptors, such as Serotonin 5-HT1A receptor; *HTR1A* (Veronese et al. 2016). Because many receptors are expressed across the whole brain, identifying a reference region that is devoid of the receptor requires pharmacological blockade. The method proposed by Veronese et al. estimates the specific and non-displaceable components of radioligand uptake based on the correlation between the abundance of the receptor gene transcript (using data from the Allen Human Brain Atlas) and the PET measurements of the expressed protein, without the need for blocking drugs.

Another promising research direction is the integration of data from the Allen Human Brain Atlas into fMRI studies to better understand the molecular mechanisms underlying functional connectivity in the human brain. One of the earliest efforts to link neuroimaging data and gene expression data in the human brain is presented by Goel et al. (2014). They explored whether structurally connected regions, those connected by white matter tracts determined by MR diffusion tensor imaging, have similar gene expression patterns as observed in rodents (French and Pavlidis 2011; Wolf et al. 2011). Despite finding no significant association between pair-wise connectivity and gene expression similarity, their results indicate that the overall connectivity of the brain is influenced by the underlying gene expression patterns. A large-scale analysis of the association between several cognitive phenomena and their underlying molecular mechanisms has been carried out in Fox et al. (2014). The study makes use of Neurosynth (Yarkoni et al. 2011), a framework to automatically synthesize brain-wide functional activation maps of cognitive tasks and psychological states based on published fMRI studies. By quantifying the spatial similarity between the expression patterns of all genes and several psychological topics, they demonstrated the ability to replicate known gene-cognition associations, such as between dopamine and reward. They further used their analysis to pinpoint previously unknown associations that can serve as a guide for researchers towards testable hypotheses about the genetic etiology of complex cognitive tasks. Cioli et al. (2014) used the Allen Human Brain Atlas to characterize the molecular differences between two sets of cortical functional networks. Using discriminant correspondence analysis, they predicted to which set of functional networks a cortical region belongs based on its gene expression profile. Richardi et al. (2015) showed that functionally connected regions, defined by a synchronized activity as measured by fMRI, are similar in their gene expression patterns compared with disconnected regions. Furthermore, they identified a set of genes underlying the relationship between correlated gene expression and functional networks, and through GO analysis, they found that these genes are significantly enriched for ion channels. Similarly, Wang et al. (2015) used a region-specific measurement of brain activity based on fMRI to identify genes that correlate with brain activity in the default mode network that is brain regions with coherent fMRI signal fluctuations at the resting state. The correlated genes were enriched in neurons as well as genes down-regulated in autism. By analyzing the relationship between genes with consistent expression patterns across individuals and resting-state functional connectivity data from the Human Connectome Project, Hawrylycz et al. (2015) suggested that functional circuits are linked to conserved gene expression patterns across the cortex. Krienen et al. (2016) analyzed the association between corticocortical functional networks and the co-expression patterns of 19 genes uniquely enriched in the supragranular layers of the human cerebral cortex, in contrast to mice. The resulting strong association of major functional cortical classes (sensory/motor, paralimbic, or associational) supports the hypothesis that this unique molecular signatures of the human upper cortical layers underlie long-distance corticocortical connections, distinguishing humans from rodents. To extend this analysis, Vértes et al. used partial least squares (Rubinov et al. 2015) to identify the transcriptional signatures associated with topological parameters of fMRI networks indicating whether cortical regions are involved in long- or short-distance connections (Vértes et al. 2016). They showed that the transcriptional profiles of hub regions are, indeed, enriched in genes specific to supragranular layers as well as genes involved in oxidative metabolism and mitochondria, supporting the high cost associated with long-distance connections.

In contrast to the aforementioned studies on integrating functional activation maps of the human brain with gene expression patterns, fewer studies analyzed the link between structural changes in MRI scans and patterns of gene expression. Whitaker et al. (2016) used MRI to study maturation of human brain structures by quantifying changes in cortical thickness and myelination throughout adolescence. To understand the molecular mechanisms underlying changes in cortical thickness and myelination at different brain regions, they analyzed the relationship between these MRI markers and gene expression patterns from the Allen Human Brain Atlas. Using a multivariate dimensionality reduction technique (partial least squares), they identified associations between the expression patterns of all genes (~20,000) and four MRI-based variables. Peng et al. (2016) investigated whether the relationships among cortical regions can be explained from genetic factors using genotype data from twins and unrelated individuals. In addition, they reported high concordance between inter-regional genetic correlations (based on genotype) and the inter-regional similarity of expression profiles using data from the Allen Human Brain Atlas, further confirming the genetic basis of cortical patterning. With the increasing interest in linking neuroimaging data to gene expression data, Rizzo et al. (2016) developed MENGA (Multimodal Environment for Neuroimaging and Genomic Analysis), which is a framework to integrate neuroimaging data from various modalities, such as PET and MRI, to gene expression patterns from the Allen Human Brain Atlas. MENGA was evaluated by analyzing the correlation between image data from different modalities focusing on the serotonin and the dopamine systems as well as myelin in brain tissue.

Romme et al. (2016) extended the study of associations between brain wiring and the underlying transcriptional signatures of connected regions to examine the role of genes in connectivity disruptions observed in schizophrenia patients. Using cross-correlation analysis of expression profiles of SCZ risk genes, identified using GWAS, and diffusion-weighted MRI, they found a strong association between the expression of the risk genes and regional macroscale dysconnectivity in schizophrenia patients. Valli et al. (2016) used the expression profiles of the glucocorticoid and mineralocorticoid receptors across the human brain to analyze the relationship between cortisol levels and gray-matter volume in individuals with ultra-high risk for psychosis. By assuming that the relationship between gray-matter volume and cortisol levels likely occurs in brain areas with high expression of cortisol receptor genes, they used an adaptive threshold to identify significant associations. These results further highlight the value of studying associations of alternations observed in brain images and the underlying transcriptional profile of the affected areas to uncover disease mechanisms as well as to identify new disease genes.

### Studying brain organization using dimensionality reduction methods

**Fig. 5:**
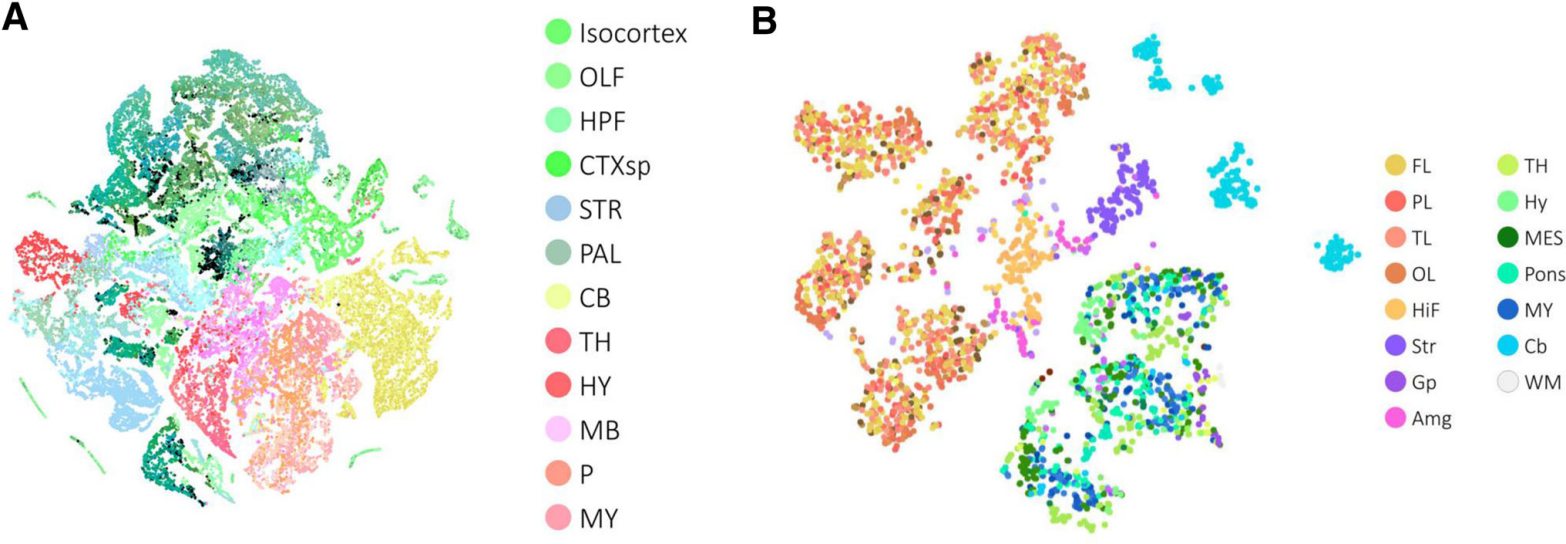
Dimensionality reduction of brain transcriptomes. Samples from brain transcriptomes can be embedded in a low-dimensional space by means of dimensionality reduction methods. **a** 2D embedding of ~60,000 voxels from the Allen Mouse Brain Atlas. **b** 2D embedding of ~3700 samples from the six donors in the Allen Human Brain Atlas. Both embeddings were generated using Barnes-Hut t-SNE. In both maps, *colors* correspond to anatomical regions of the mouse and human brain. Data from Mahfouz et al. (2015a)

An alternative approach to analyze the relationship between gene expression and neuroanatomy is dimensionality reduction (Box 2). Mapping high-dimensional data in two dimensions allows for the exploration of how gene expression patterns relate to brain organization. Ji (2013) used t-distributed stochastic neighborhood embedding (t-SNE) to map the Allen developing mouse brain atlas and showed that t-SNE clearly outperforms PCA. The results show that clustering voxels in the low-dimensional space is more consistent with neuroanatomy than in the original space. Mahfouz et al. (2015a) used a computationally efficient implementation of t-SNE, named Barnes-Hut-SNE, to map the sagittal and coronal adult mouse atlas and the brain transcriptome of the six human donors (Fig. 5). They quantitatively showed that BH-SNE maps are superior in their separation of neuroanatomical regions in comparison to PCA and MDS. Similarly, dimensionality reduction approaches can be used to analyze the gene–gene relationships. A low-dimensional embedding of genes in which distances represent similarity of the spatial and/or temporal expression profile of genes across the brain can be very informative.

## Perspective on the future of computational analysis of brain transcriptomes

### Brain transcriptome atlases are no cell-type-specific

The identification of the molecular profile of the different cell types in the brain, their connectivity patterns, and their electrophysiological properties is crucial to our understanding of the functional organization of the brain. Despite the valuable information provided by the brain transcriptomes, these resources remain limited in their ability to quantify cell-type-specific expression of genes. New technologies targeting specific cell populations, such as viral, optogenetic and single-cell sequencing approaches, will allow us to better characterize cell types and their role in brain function. So far, these techniques are limited in their scalability and computational methods still provide a feasible alternative approach. Using spatial clustering of gene expression patterns of cell-type-specific genes in the adult mouse, Ko et al. (2013) showed that astrocytes and oligodendrocytes differ between brain regions, but these regional differences in expression are less pronounced than differences in neuronal composition. Similarly, Grange et al. (2014) proposed a model to estimate cell-type-specific maps of the mouse brain. Kuhn et al. (2011) developed a method to analyze brain samples of varying cellular composition. Their method detected myelin-related abnormalities in brain samples from Huntington’s disease patients, which was not detected using standard differential expression. These examples illustrate the power of computational models in untangling the complex composition of the different cell types in the brain.

With the recent advances in single-cell mRNA sequencing, it has become feasible to measure the expression of thousands of genes and their variability between different cell types (Shapiro et al. 2013). In addition, single-cell sequencing has indicated that neurons from small cortical regions come from different clones with distinct somatic mutations (Lodato et al. 2015). Understanding how these different clones of neurons contribute to the aggregated gene expression from a specific brain region will be of great interest to understand the role of mutations in neurological disorders. The vast amount of data generated by these projects illustrates the importance of computational methods that can identify distinct groups of cells with a common functional role (Pettit et al. 2014; Grün et al. 2015).

### Limited resolution of brain transcriptomes

There are several limitations associated with the current spatial and temporal brain transcriptomes. Despite their unprecedented spatial and temporal resolution, human brain transcriptomes are still of low resolution with ~1000 samples per brain. This relatively low resolution presents a fundamental limitation, especially when integration with imaging-based data (e.g., MRI or PET) is considered. The ISH-based mouse transcriptomes offer a much higher resolution. Although the original ISH data provide a near-cellular resolution (~1 µm), the genome-wide data registered to the common 3D space offer a much lower resolution (~200 µm). Several studies used re-registration of a limited set of the high-resolution ISH images from the Allen Mouse Brain atlas to acquire genome-wide data at a higher resolution. The aforementioned study by (Ko et al. 2013) found more transcriptionally distinct brain regions than a previous study (Bohland et al. 2010), mainly due to the usage of cell-type specific genes. However, Ko et al. have also realigned the ISH images of the mouse brain atlas and performed their analysis on a higher resolution grid (100 µm). Ramsden et al. (2015) used non-linear registration to realign the ISH data of the mouse. By analyzing genome-wide data at a resolution of 10 µm, they were able to identify genes whose expression pattern delineates the borders and layers of the medial entorhinal cortex.

There is still need for more generic approaches to map spatially mapped gene expression data (from ISH experiments) generated at different labs to the standard 3D space of the Allen Reference Atlas. Tools, such as BrainAligner (Peng et al. 2011), are available for analyzing *Drosophila melanogaster* neural expression patterns. The availability of similar tools for the mouse and human brain can enormously enhance our understanding of disease molecular mechanisms by allowing researchers to map their own data to the same space.

### Current atlases focus only on protein-coding mRNA

Most of the atlases profiling the mammalian brain transcriptome and its relationship to brain development and function have mainly focused on profiling the expression of protein-coding mRNA. These atlases mostly provided limited or no information about other RNA species, such as non-coding RNA (ncRNA) and microRNA (miRNA), despite their recognized role in brain development and neurological disorders (Ponjavic et al. 2009; Qureshi and Mehler 2012). Using the Allen Mouse Brain Atlas, long ncRNAs showed regionally enriched expression patterns, such as those observed for protein-coding mRNAs (Mercer et al. 2008), further supporting their functional role in the brain. By profiling the developmental transcriptome of the neocortex using deep sequencing, Fertuzinhos et al. defined the dynamics of mRNA, miRNA, and ncRNA across the different layers of the mouse cortex (Fertuzinhos et al. 2014). The BrainSpan atlas provides the most comprehensive map of miRNA expression in the developing human brain. Ziats and Rennert (2013) used the BrainSpan miRNA data to define a pattern of increased inter-regional expression differences of miRNA through development, potentially driving regional specialization. Moreover, targets of differentially expressed miRNAs were mostly related to transcriptional regulation and neurodevelopmental disorders, highlighting the importance of studying miRNAs as potential biomarkers. Additional measurement of ncRNAs and miRNAs as well as a detailed analysis of their role in gene regulatory networks can help our understanding of their relationship to genes related to neurodevelopmental disorders.

### Integrating brain transcriptomes and other neuro-omics data sets

Advances in high-throughput molecular profiling have facilitated acquiring various omics data sets spanning a wide spectrum of cellular processes. For instance, the rapid developments in next-generation sequencing (NGS) technology enabled genome-wide measurement of genomic, transcriptomic, and epigenomic data of brain tissues. While transcriptomes provide detailed information on the abundance of RNA, epigenomic features, such as histone modifications, methylation, and chromatin interactions, describe the underlying mechanisms of distinct cell-specific transcriptomes. Moreover, most disease-related variants are in the non-coding regulatory regions of the genome, making epigenomic studies crucial to uncover a larger proportion of the genetic contribution to complex traits than can be explained by coding variants alone. Increasingly, studies are gathering data across different platforms from a wide range of tissues and cell types to uncover mechanisms underlying complex phenotypes and disease. The Encyclopedia of DNA Elements (ENCODE) (Bernstein et al. 2012a) and the Roadmap Epigenome project (Consortium 2015a) have profiled the epigenome of several tissues and cell types, while the Genotype Tissue Expression project (GTEx) (Lonsdale 2013) is generating genotype and gene expression data from 25 unique human tissues, including 13 brain regions. In addition, The Cancer Genome Atlas project (TCGA) (Weinstein et al. 2013) and the International Cancer Genome Consortium (ICGC) (Hudson et al. 2010) provide comprehensive genomic and transcriptomic and epigenomic data from multiple cancer types. However, most of these studies have profiled samples from cancer cell lines or normal cells from non-brain tissues due to limitations specific to the brain, such as the requirement of large amount of genomic material and the high heterogeneity of cell types within the same sample (Shin et al. 2014). Recently, the isolation of more homogeneous samples from the brain as well as developments in single-cell analysis is greatly advancing the field of neuroepigenomics (Maze et al. 2014; Shin et al. 2014). For example, efforts have been made to map the brain methylome (Illingworth et al. 2015) and to identify *cis*-regulatory elements across brain regions (Vermunt et al. 2014). The PsychENCODE consortium (Akbarian et al. 2015) is an ongoing project to profile neurobiological epigenetic landscape of the healthy and diseased developing and adult human brains. For large-scale multi-omics data sets, systems genomics approaches, which integrate different genome-wide data types, can minimize false positive discoveries as well as unravel the molecular mechanisms underlying the phenotype or disease of interest. Several approaches have been developed to integrate multi-omics data (Consortium 2015b), clearly illustrating the added value of collecting multiple omics measurements from a large number of samples.

### Integrating brain transcriptomes and imaging mass spectroscopy

Over the past few years, imaging mass spectrometry (IMS) (Caprioli et al. 1997) has emerged as a powerful technique to capture the spatial distribution of large biomolecules, such as proteins, peptides, and lipids in biological samples. Similar to ISH, imaging mass spectroscopy holds great potential in studying the chemical organization of complex samples from the brain (Hanrieder et al. 2013). Methods have been developed to align IMS-based sections of the mouse brain to histology-based sections from the Allen Mouse Brain Atlas to anatomically localize biomolecules within the brain (Abdelmoula et al. 2014; Carreira et al. 2015). However, recently, these methods have been extended to link protein expression to the expression of the encoding gene as well as its co-expressed genes based on the Allen Mouse Brain Atlas (Škrášková et al. 2015). There is a great potential for applications based on the integration of ISH-based gene expression and IMS-based protein expression measurements to help our understanding of translational mechanisms in the brain. Yet, more complex modeling of the two data types is needed. Methods developed to integrate spatially mapped gene and protein expression data can also be used to study spatial localization within the cell using data from the Human Protein Atlas (Uhlen et al. 2015).

### Imaging genetics

In an attempt to better understand gene-disease associations, researchers are searching for genes that affect intermediate disease biomarkers. Brain imaging studies can be used to reveal genetic effects on brain structure, function, and circuitry, providing valuable mechanistic insights. Imaging genetics have emerged as a field concerned with finding associations between genetic variants (typically SNPs) and imaging-based measurements (Hibar et al. 2011b). Due to the millions of statistical tests that need to be performed, stringent statistical thresholds are required to limit the false discovery rate (Medland et al. 2014). Recently, the Enhancing Neuro Imaging Genetics through Meta-Analysis (ENIGMA) consortium (Hibar 2015) analyzed SNPs associations with the volume of sub-cortical structures in ~30,000 individuals, providing the first large-scale analysis of the genetic causes of human brain variability. Several methods have been developed to limit the number of statistical tests performed in genome-wide, brain-wide analysis by either exploiting the dependency between brain voxels and/or testing for associations with genes or pathways instead of individual variants (Hibar et al. 2011a). In addition, efforts have been made to jointly model imaging and genetic observations from Alzheimer’s Disease Neuroimaging Initiative (ADNI) data (adni.loni.ucla.edu), using multivariate statistical methods (Wang et al. 2012; Batmanghelich et al. 2013). These methods remain computational very expensive, limiting the number of variables analyzed. Brain transcriptomes can play an important role in imaging genetics by providing region-specific information about gene expression that can be used to prioritize genes and variants for testing. For example, incorporating spatial gene co-expression of amyloid-related candidate genes from the Allen Human Brain Atlas as prior knowledge to their statistical model significantly improved the prediction of associations between SNPs in the APOE gene and amyloid deposition measures among cortical regions (Yan et al. 2014). There is need for more advanced methods to link genomic measurements which are usually collected from blood samples to intermediate disease phenotypes observed in brain images.

### Unexplored computational avenues

The multiple dimensions of the brain transcriptomes (genes, regions, and time) provide a framework to explore spatio-temporal regulation of gene expression during development. Clustering the data along one dimension only yields global patterns of similarity, while in a complex system, such as the brain, it is always more useful to identify more localized patterns of correlation. For example, the effect of steroid hormones on the brain is highly region-specific, depending on the availability of target genes and co-regulators affecting the steroid receptors at the site of action. Analyzing the region-specific co-expression relationships of steroid receptors and their coactivators can be used to predict steroid responsiveness and selective activation of particular circuits with synthetic ligands (Zalachoras et al. 2013).

Biclustering is a type of technique to simultaneously identify a subset of genes associated with a subset of conditions (this can be brain regions and/or time-points), allowing for the identification of local spatial or temporal patterns of co-expression. Biclustering has been particularly effective in analyzing time-series expression data (Goncalves and Madeira 2014). Similarly, applying biclustering to expression data from the Allen Mouse Brain Atlas resulted in more GO-enriched clusters than those obtained by independently clustering genes or regions (Jagalur et al. 2007). Ji et al. (2013) described a co-clustering method based on graph approximation to explore the spatio-temporal regulation of gene expression during the mouse brain development. Yet, they apply biclustering to each developmental stage independently and do not consider the time-varying nature of the developing mouse brain data, due to the lack of correspondence between the voxels across different stages. To fully exploit the multi-dimensionality of the developing brain transcriptomes, triclustering methods provide an interesting approach to identify groups of genes that show spatial and temporal co-expression (Tchagang et al. 2012). Recently, Jung et al. (2015) used three-component analysis to identify genes associated with aging by analyzing longitudinal gene expression, methylation, and histone modification data of human skin fibroblasts. Their three-component analysis represents an integrative approach to jointly model temporal changes in different data types. An extension of their methods to incorporate spatial information available in brain transcriptomes can lead to a complete approach of modeling spatial and temporal changes of different omics data from the brain.

Graphical models (e.g., conditional random fields) are commonly used for data segmentation using local features, especially in computer vision application. The Roadmap Epigenome project has used a Hidden Markov Model to classify the human genome into chromatin states based on epigenetic markers (Consortium 2015a). These models can be used to model the spatial and/or temporal relationships between genes in brain transcriptome atlases.

A greater challenge lies in identifying causal relationships rather than associations in gene–gene interactions and the brain is no exception. Systems biology approaches provide an interesting avenue to explore causal relationships between genes by means of quantitative modeling. The resulting mathematical models enable formal analysis and simulation of complex biological processes (Kolch et al. 2015). However, inferring causal relationships between the different variables requires a vast amount data, limiting their usability to a small number of genes (Lausted et al. 2014). Hwang et al. (2009) presented a system approach to analyze genes differentially expressed in the mouse brain across time in Prion disease. An extension of such a model to include spatial information on gene expression can help refine the model as well as associate disease-related changes to specific brain areas.
